# Water warming increases aggression in a tropical fish

**DOI:** 10.1038/s41598-020-76780-1

**Published:** 2020-11-18

**Authors:** Zi Xun Kua, Ian M. Hamilton, Allison L. McLaughlin, Reed M. Brodnik, S. Conor Keitzer, Jake Gilliland, Elizabeth A. Hoskins, Stuart A. Ludsin

**Affiliations:** 1 Aquatic Ecology Laboratory, Department of Evolution, Ecology, and Organismal Biology, The Ohio State University, 1314 Kinnear Road, Columbus, OH 43212 USA; 2 Department of Evolution, Ecology, and Organismal Biology, The Ohio State University, 318 W 12th Avenue, Columbus, OH 43210 USA; 3 Department of Mathematics, The Ohio State University, 318 W 12th Avenue, Columbus, OH 43210 USA; 4 Present Address: Department of Sustainable Resources Management, College of Environmental Science and Forestry, State University of New York, 1 Forestry Drive, Syracuse, NY 13210 USA; 5 Present Address: Department of Biology, University of Kentucky, 101 T.H. Morgan Building, Lexington, KY 40506 USA; 6 Present Address: Chesapeake Biological Laboratory, University of Maryland Center for Environmental Science, 146 Williams St., Solomons, MD 20688 USA; 7 Present Address: Department of Natural Science, Tusculum University, Greenville, TN 37745 USA; 8 Present Address: Nationwide Children’s Hospital, Columbus, OH 43205 USA

**Keywords:** Climate-change ecology, Behavioural ecology, Animal behaviour

## Abstract

Our understanding of how projected climatic warming will influence the world’s biota remains largely speculative, owing to the many ways in which it can directly and indirectly affect individual phenotypes. Its impact is expected to be especially severe in the tropics, where organisms have evolved in more physically stable conditions relative to temperate ecosystems. Lake Tanganyika (eastern Africa) is one ecosystem experiencing rapid warming, yet our understanding of how its diverse assemblage of endemic species will respond is incomplete. Herein, we conducted a laboratory experiment to assess how anticipated future warming would affect the mirror-elicited aggressive behaviour of *Julidochromis ornatus*, a common endemic cichlid in Lake Tanganyika. Given linkages that have been established between temperature and individual behaviour in fish and other animals, we hypothesized that water warming would heighten average individual aggression. Our findings support this hypothesis, suggesting the potential for water warming to mediate behavioural phenotypic expression through negative effects associated with individual health (body condition). We ultimately discuss the implications of our findings for efforts aimed at understanding how continued climate warming will affect the ecology of Lake Tanganyika fishes and other tropical ectotherms.

## Introduction

Human-induced rapid environmental change (HIREC)^1^ has altered species interactions and caused species range shifts, leading to population declines in both aquatic and terrestrial ecosystems^2–7^. Novel physical conditions caused by HIREC, which exert selective pressures that have not been previously encountered during an organism’s evolutionary history, are expected to be especially detrimental to species persistence^1,8,9^. While the literature is rich with examples of how novel selection pressures caused by HIREC, including habitat destruction^10,11^, pollution^12^, over-exploitation^13,14^, and species invasions^15,16^ have caused population declines, we are only beginning to fully appreciate the impact of climate-driven changes to the environment.

Our expectation is that the impact of climate change will vary among the world’s biomes, with species that have evolved in thermally stable, tropical environments being more vulnerable to climate change than temperate species that have evolved in more thermally variable environments^17–19^. In this way, the conventional wisdom is that even small changes in the climatic regime (e.g., warming) would have large effects on the species that inhabit coral reef, river, and forest ecosystems in the tropics^4,9,20–22^. Furthermore, changes in the thermal environment are expected to affect the demographics of native populations through direct and indirect influences associated with altered physiology (e.g., metabolism)^23,24^.

For aquatic ectotherms such as fish residing in tropical lakes, warming could be problematic for at least two reasons. First, increased water temperature is expected to raise basal metabolic rates, which might push these organisms past their optimal thermal window^18^. In turn, their need to acquire more energy to survive, grow, and reproduce would be expected to increase^25–27^. Second, because warming can alter the thermal structure of lakes such that primary production decreases^28–30^, insufficient energy may be available to support the production of prey needed to counterbalance the expected increase in metabolic demand.

While species can and have evolved to cope with environmental changes^31,32^, rapid warming and other forms of HIREC likely will require alternative means for populations to survive under these kinds of selection pressures. Behavioural change offers a mechanism by which individuals could persist in the face of rapid environmental change. For example, individuals may become bolder and more aggressive towards conspecifics as a means to secure food, territorial, and reproductive resources^33–35^. The effect(s) of HIREC on individual behaviour unfortunately remain(s) less understood than other aspects of an organism’s phenotype (e.g., physiological responses)^36^.

Understanding this linkage is important because changes in an individual’s behavioural phenotype hold great potential to alter community (food web) interactions, as well as mediate future fitness. Increased boldness or aggression in fish, for instance, has been shown to increase the risk of being preyed upon by higher consumers^37^, as well as being harvested by human fishers^38–40^. While previous studies have shown that rapid warming can cause an acute (immediate) increase in aggression in ectotherms such as fish^35,41–43^, whether this effect on behavioural phenotype is chronic (long-lasting) remains unclear, owing to the potential for acclimation.

This knowledge gap could be especially important for understanding the dynamics of organisms that form pair bonds or reside in social groups. Pair bonding and group-living are common in many animals, owing to the benefits that they can confer to activities vital to individual fitness, including territory defence^44,45^, care of young^46,47^, and safety from predators^48,49^. Crucially, the stability and reproductive success of social groups, including mating pairs, can be influenced by the behaviour (e.g., aggression) of individuals, as has been shown for the highly social Lake Tanganyika cichlid *Neolamprologus pulcher*^50^. Temperature-induced changes in behaviour, therefore, could result in fitness effects through both the direct consequences of individual behaviour (e.g., risk of injury from increased aggression) and indirect impacts of changes in an individual’s social context^41^.

The need for such knowledge is especially evident in Lake Tanganyika, a Great Lake located in eastern Africa. As with other African Rift Lakes, Lake Tanganyika supports a flock of endemic (cichlid) fishes that is being threatened by HIREC, including climate warming^51–54^. In addition to potentially raising the metabolic demand of these ectothermic fish^27,55^, observed rapid increases in surface (epilimnetic) water temperature during recent decades has strengthened thermal stratification^28–30,56–58^. In turn, both the upwelling of nutrient-rich bottom waters and vertical mixing of the water column have decreased, resulting in reduced productivity of the lower food web that resides in the epilimnion^56,59^. Because thermal stratification is expected to only strengthen and last longer with continued warming of epilimnetic waters^30^, the need to understand how organisms that reside in epilimnetic waters of this ecosystem will respond to continued warming is paramount^58,60^.

Herein, we conducted a laboratory experiment with an endemic Lake Tanganyika cichlid, *Julidochromis ornatus* (golden julie), to better understand how anticipated water warming might affect individual behaviours over the long-term within this species and potentially the many other endemic cichlids like it. We chose *J. ornatus* as our study species because it has a life-history similar to the many other endemic cichlids in East Africa and it is commonly found in the shallow, nearshore areas of Lake Tanganyika, have been vulnerable to water warming^56,58,60^. Specifically, we exposed *J. ornatus* adults to one of two thermal conditions: current-day water temperatures (~25.5 $^\circ$C)^56,61^ and the projected water temperature at the end of the century (~29 $^\circ$C)^56,58^, which has already been observed in surface waters of Lake Tanganyika^58,61^.

To increase realism in our experiment and to assess the potential for long-lasting behavioural change, we used longer initial laboratory acclimation (10–12 months), thermal transitional (2 weeks), and experimental exposure (8 months) periods than other studies of this kind^35,41–43^. We predicted that individuals in the high-temperature (i.e., future climate) treatment would become more aggressive relative to the low-temperature (i.e., control) treatment, because (1) temperature and metabolic rate have been shown to be positively correlated in ectotherms^18,62^ and (2) other studies with ectotherms have generally shown individual metabolic rate and aggression to be positively related^36,37,55,63^.

## Results

### Body size and condition

Differences in body size and condition became evident during the course of the experiment, which needed to be accounted for in our predictive modelling of aggression. No significant differences in mean wet mass (*M*), total length (*TL*), and body condition (*cond*) existed between the low-temperature and high-temperature treatments at the start of the experiment (all $\chi ^2_{1,102} < 0.6$, $p>0.4$). However, significant size differences existed 17 months later, at the experiment’s conclusion (for both *M* and *TL*: $\chi ^2_{1,102}>44$, $p<0.001$). Specifically, fish grew from a mean ($\pm 1$ SD) size of $2.6\pm 1.1$ g and $57.4\pm 7.6$ mm at ~ 9 months of age to $3.4\pm 1.4$ g and $62.3\pm 8.3$ mm at 26 months of age; we reported pooled means across treatments because neither temperature nor its interaction with time (Pre vs. Post) explained a significant amount of variation in *M* or *TL* (both $\chi ^2_{1,102} < 0.6$, $p>0.3$). By contrast, a statistically significant change in *cond* was not observed during the course of the experiment ($\chi ^2_{1, 102}=0.58$, $p>0.4$).

Given that fish size and health have been shown to affect individual behaviour^36^, as well as the fact that significant somatic growth occurred during the 8 months of experimentation and more than 2-fold individual variation in *M* and *cond* was observed within and among treatments (*M* range = 0.9–5.7 g; *cond* range = 1.9–4.2), we retained both *M* and *cond* as covariates in our aggression analyses. While *TL* also varied considerably within and among treatments (*TL* range = 43.2–75.0 mm), we did not include this factor as a potential covariate in our modelling to reduce multicollinearity, given that *TL* was highly correlated with *M* ($r=0.95, p<0.001, df = 100$).

### Aggression

Aggression scores varied widely during the course of the experiment, with a wide range of aggressive displays, both overt and restrained, being observed (Supplementary Fig. S1). Because overt and restrained aggression were significantly positively correlated ($r = 0.36, p<0.001, df = 100$), and total aggression was positively correlated with both overt and restrained aggression scores (both $r > 0.8, p<0.0001, df = 100$), we used total aggression as our main response variable in subsequent analyses.

Our model selection procedure identified a single predictive model of total aggression as the most robust (AICc = 1125.8; weight = 0.46; Table 1). This model consisted of only temperature (*temp*; $F_{1, 101}=14.84$, $p<0.001$) and the temperature$\times$time interaction (*temp:time*; $F_{1,101}=10.80$, $p<0.01$) as predictors. However, body condition (*cond*; $F_{1, 101}=9.23$, $p<0.01$) and test rank ($F_{1, 101}=4.36$, $p<0.05$) were also included in the final model as important covariates (Table 1).

**Table 1:** Top five linear models from the Akaike information criterion (AICc) comparison of all possible iterations of the global model (Global model: Agg = Temp * Time * Sex + ln(Mass) + Cond + Test Rank), used to identify the most parsimonious model to explain changes in *Julidochromis ornatus* aggression.

Model	(Intercept)	Cond	Log(Mass)	Sex	Temp	Test rank	Time	Sex:temp	Sex:time	Temp:time	Sex:temp:time	Df	logLik	AICc	Delta	Weight
**1**	**349.84**	**− 50.70**	**NA**	**NA**	**+**	**− 12.90**	**+**	**NA**	**NA**	**+**	**NA**	**7**	**− 555.29**	**1125.78**	**0.00**	**0.46**
2	352.00	− 52.09	NA	+	+	− 13.54	+	NA	NA	+	NA	8	− 555.16	1127.87	2.09	0.16
3	332.74	− 53.69	NA	NA	+	NA	+	NA	NA	+	NA	6	− 557.56	1128.00	2.23	0.15
4	351.21	− 49.82	− 4.44	NA	+	− 12.83	+	NA	NA	+	NA	8	− 555.23	1128.01	2.23	0.15
5	343.65	− 51.07	NA	+	+	− 13.40	+	NA	+	+	NA	9	− 554.61	1129.18	3.41	0.08
Global	351.81	− 50.27	− 9.55	+	+	− 14.00	+	+	+	+	+	12	− 554.09	1135.70	9.92	0.00
Null	189.43	NA	NA	NA	NA	NA	NA	NA	NA	NA	NA	2	− 570.08	1144.28	18.51	0.00

Akaike coefficient values are shown for continuous variables; “+” indicates inclusion; “NA” indicates omission; $\Delta$AICc > 2.0 indicates a difference; “Agg” indicates aggression scores calculated from behavioural assessment; “Temperature” indicates low- and high-temperature treatment (“Low” = 25.5 $^\circ$C; “High” = 29 $^\circ$C); “Time” indicates pre- and post-treatment period (“Pre” = 9–18 months of age; “Post” = 18–26 months of age); “ln(Mass)” indicates the natural log-transformed wet mass of individual fish; “Cond” indicates scaled-mass condition index; and “Test Rank” indicates the relative numeric order of the behavioural trial of the same day and experimental aquarium. Results for the null and global models are also presented. The most parsimonious model is in bold-face font.

**Figure 1:**
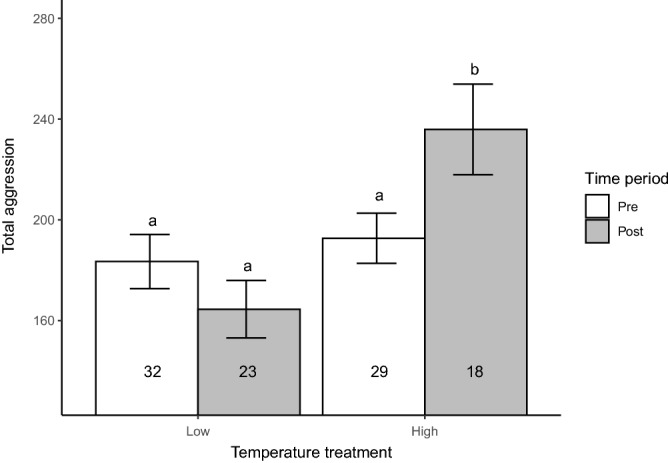
Total aggression scores of *Julidochromis ornatus* at the start (Pre; measured at 9 months of age) and end (Post; measured at 26 months of age) of our laboratory experiment. While all individuals were subjected to 25.5 $^\circ$C (Temperature = Low) prior to the start of the experiment (Time = Pre; 9 to 18 months), temperature was raised to ~29 $^\circ$C during 18–26 months of the experiment (Time = Post) in half of the tanks (Temperature = High), whereas it remained unchanged in the other half of the tanks (i.e., Temperature = Low). The mean (bars) and standard errors (error bars) of total aggression in each treatment are presented. Numbers in each bar indicate sample sizes. Treatments sharing a letter do not significantly differ (Tukey-adjusted *LSMeans* comparisons; $p < 0.05$).

Increased temperature led to increased aggression in our experiment (Table 2; Fig. 1). While total aggression scores did not significantly differ between treatments before the temperature manipulation, total aggression differed between treatments after the temperature manipulation (Table 2; Fig. 1). Specifically, total aggression scores significantly increased for the individuals held in the manipulated (high-temperature) treatment (ranged: ~194 to ~247 in a 10-min trial), whereas it remained unchanged in the individuals held in the control (low-temperature) treatment (Table 2; Fig. 1).

**Table 2:** Pairwise comparison of the least-squares means (LS means) and associated standard errors (SE) of *Julidochromis ornatus* total aggression scores by temperature (“Low” = 25.5 $^\circ$C; “High” = 29 $^\circ$C) and time (“Pre” = 9–18 months of age; “Post” = 18–26 months of age).

Temperature	Time	LSmean	SE	Df	Lower CL	Upper CL	Group
Low	Pre	181.40	10.22	96	155.46	207.33	a
High	Pre	193.97	12.04	96	163.41	224.53	a
Low	Post	157.82	10.89	96	130.17	185.47	a
High	Post	248.85	14.05	96	213.18	284.53	b

Means sharing a letter in “Group” column do not significantly differ (Tukey-adjusted LS means comparisons; $p<0.05$). Df indicates degrees of freedom. The lower and upper confidence limit (CL) of the means are also presented.

**Figure 2:**
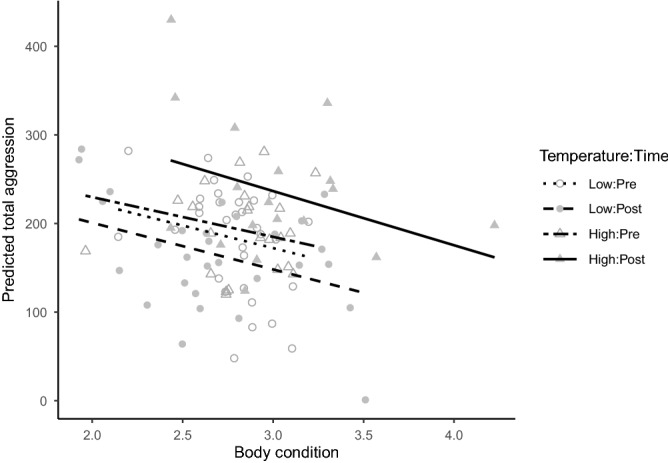
Predicted total aggression scores using the most parsimonious (based on AICc) linear mixed model versus body condition in *Julidochromis ornatus* individuals used in our laboratory experiment. All individuals were subjected to 25.5 $^\circ$C (Temperature = Low) prior to the start of the experiment (Time = Pre; 9 to 18 months). By contrast, temperature was raised to 29 $^\circ$C (Temperature = High) during 18–26 months of the experiment (Time = Post) in half of the tanks (Temperature = High), whereas it remained unchanged in the other half of the tanks (i.e., Temperature = Low). The regression line for each treatment is shown.

While the effect of test rank (order) on aggression was inconsistent in relation to total aggression (Supplementary Fig. S2), the seeming effect of body condition on total aggression was clearer. In general, aggression was inversely related to body condition, as indicated by the negative coefficient (-50.7) associated with *cond* in our selected predictive model (Table 1). To further illustrate this relationship, we used our most parsimonious model to predict total aggression as a function of body condition in all treatments (Fig. 2). This analysis showed that, even though individuals from the high-temperature treatment had an overall greater mean ($\pm 1$ SE) condition at the end of the experiment ($3.0\pm 0.1$) compared to the individuals in the low-temperature treatment ($2.7\pm 0.1$), *cond* was negatively related to aggression. Thus, individuals in poorer condition were generally more aggressive than those in better condition regardless of treatment (Fig. 2).

## Discussion

The main objective of our study was to shed insight into how climate warming might affect the behaviour of tropical freshwater ectotherms such as the endemic Lamprologini cichlid assemblage of Lake Tanganyika. Three major findings emerged from our experiment. First, we found that the frequency of aggressive behaviour in the Tanganyikan cichlid *Julidochromis ornatus* increased after individuals were exposed to the future projected temperature of Lake Tanganyika surface waters at the end of the 21^st^ century (i.e., ~29 $^\circ$C)^56^, which also happens to be the current-day extreme high temperature^56,61^. The finding is important because the observed increase in aggression with increased water temperature indicates that this behaviour is not canalized with respect to temperature for this species, which may help this (and perhaps other tropical) species persist in the face of continued human-induced rapid environmental change (HIREC). Second, our experiment showed that environmental change such as water warming can modify the behaviour of ectotherms like fish, not only in the short-term as other studies have previously documented^35^ but also over the long-term; increased aggression persisted for the duration of our 8-month experiment, which we would expect to have major consequences to individual energy budget, growth, and fitness, as well as community and social interactions. Finally, we unexpectedly found that individual aggression in *J. ornatus* adults increased as individual body condition (our proxy for health^36^) declined. Thus, individuals in the poorest condition were more likely to expend additional energy exhibiting aggressive behaviours than those in the best condition. Such behaviour could be viewed as highly risky, with potentially negative consequences for future growth performance and fitness, in the absence of sufficient prey resources to sustain this energetically expensive behaviour^55,64,65^. Below, we discuss these findings in more detail, as well as the implication of this behavioural shift in ecosystems such as Lake Tanganyika, where climate warming has also been causing reduced production at the base of the food web in shallow surface waters where cichlids such as *J. ornatus* reside^28–30,60^.

Our experiment clearly demonstrated the ability of environmental warming to increase aggression, a finding that has been documented in other ectotherms, including other fish^34^, amphibians^66^, and arachnids^67^. Currently, however, the proximal mechanism driving this linkage still remains uncertain. For example, we do not know whether this relationship is related to altered physiology (e.g., increased metabolism), behavioural “stress” associated with lost reproductive output, or perhaps just a spurious response associated with another behavioural and (or) physiological change not measured in our experiment.

At the outset of this experiment, we expected aggressive behaviour to increase in our high-temperature treatment, given that temperature and metabolic rate have been shown to be positively correlated in ectotherms^18,62^ and other studies with ectotherms have demonstrated positive relationships between individual metabolism and aggression^36,37,55,63^. However, whether environmentally-driven metabolic change can truly drive behavioural change still remains largely theoretical^68^, which is partly due to the complexity of processes that govern behavioural changes^36^ and partly due to ambivalent support from the literature. For example, individuals with a fast standard metabolic rate have been shown to be inherently more aggressive and bolder (i.e., have a fast pace-of-life) than conspecifics with a slow standard metabolic rate^69^. Likewise, contrary results have been found, wherein bolder individuals had a lower standard metabolic rate than less bold ones^70^.

Although we cannot be certain, we suspect that the increase in aggression in the individuals exposed to the higher temperature was driven by changing metabolic demands. Similar to other fish taxa^71–74^, as well as other ectotherms^75–78^, metabolic rate has been shown to increase with increasing temperature in *J. ornatus*^27^ and other cichlids^55^. For example, using a similar approach to assess aggression as our own, overt aggressive behaviours associated with locomotion (e.g., biting, tail-slapping) in *Oreochromis mossambicus*, another African cichlid, were found to be positively correlated with metabolic rate^55^. Furthermore, in a study conducted with a small subset of adult males from our study population (*n* = 8 and *n* = 10 in the low- and high-temperature treatments, respectively), those individuals exposed to the elevated temperature (29 $^\circ$C) appeared incapable of physiologically acclimating, as their routine metabolic rate significantly increased relative to the pre-manipulation period and also was significantly higher than those individuals in the control treatment (25 $^\circ$C), after 6 months of exposure to the temperature manipulation^27^. Additionally, the adult males in the high-temperature treatment did not gain (or lose) body mass, whereas individuals in the low-temperature treatment gained mass, with breeding pairs associated with males in the high-temperature treatment experiencing a significantly greater reduction in their mean reproductive rate (e.g., average number of broods produced per pair per day)^27^.

While we do know that mass-specific metabolic rates did not differ among our study individuals across our four recirculating systems at the outset of experiment (i.e., pre-temperature manipulation; R.M.B. and S.A.L., unpublished data), insufficient metabolic data existed at the end of the experiment to allow us to determine the role of metabolic change in driving aggression. Thus, we do not know if these results are generalizable to our (broader) study population. Given this uncertainty, more work is needed to help us better understand whether, for example, increased metabolic rate itself, hunger associated with increased metabolic needs, some other physiological change such as altered stress hormone levels, lost reproductive fitness, or their combination underlies the long-lasting effect that increased temperature had on aggression. In addition to having the metabolic data, knowing the time-course of aggression (i.e., when it first emerged) and stress levels of fish (e.g., corticosteroid levels)^79^ would have been helpful in delineating the proximal mechanism(s).

Even though aggression in African cichlids like *Julidochromis ornatus* is common in nature^80,81^, our observed long-lasting increase in aggression suggests that this behaviour is not canalized. This finding is important as it indicates the potential for this behavioural adjustment to help buffer this species, and perhaps others like it, against environmental change such as water warming. The ability to become more aggressive might allow individuals to better protect and obtain resources^82–84^, which may become limiting in Lake Tanganyika, if climate change continues to reduce primary production in shallow surface waters^28–30^. Heightened aggression could allow organisms to take over and control better territories, which could provide enhanced access to resources^85–87^ that can benefit both growth^88^ and survival^89^. We would also expect more aggressive individuals to secure food more readily than their less-aggressive counterparts, perhaps allowing the aggressors to outgrow and outcompete other individuals^90–93^. If true, innately aggressive individuals, as well as those with an ability to adjust their behaviour to become more aggressive, might be expected to have a fitness advantage over non-aggressive individuals and those that lack behavioural plasticity in tropical ecosystems like Lake Tanganyika, which are experiencing climate-driven water warming and reduced production at the base of the food web^28,29,58^.

In reality, however, such potential behavioural benefits are more nuanced. Heightened aggression, for example, could expose individuals to a higher risk of mortality from predators^83,94^. Likewise, while we would expect the more dominant and aggressive individuals to control better breeding shelters in nature, the corresponding rewards of territory control are likely to depend on the territory’s function in conjunction with individual’s general foraging habits. Aggressive individuals in some species (e.g., *J. ornatus*) may still struggle to find sufficient food resources to sustain their heightened metabolism and activity level, if their breeding territory does not also help them secure food resources. In this way, the heightened, energetically expensive^55,64,65^ aggressive behaviours displayed by individuals in poorer condition (health) could exacerbate the negative impacts of temperature increase. Because our study design did not allow us to explore intraspecific interactions, we see value in continued laboratory and field-based research endeavors aimed at determining whether aggression serves as a net benefit or cost to individuals under increased thermal regimes. Equally as interesting would be learning how size, sex, and body condition interact to affect the acquisition of resources such as breeding territories and food under different thermal regimes. The need to understand the costs and benefits of temperature-driven increases in aggression is especially important for Lamprologini cichlids, given that climate change has been interacting with other forms of HIREC in Lake Tanganyika to reducing production at the base of the lake’s food web^28–30^.

Although exceptions exist^95^, exposure to food shortages have been shown to generally increase aggression in other species^38,82,86,96–98^, including ectotherms^93,99^. Thus, our finding that surviving pairs in our high-temperature treatment were generally more aggressive than those in our low-temperature treatment is supported by the literature and hints at behavioural adjustment or plasticity as a means to cope with stress (e.g., potential food shortages) caused by water warming or other forms of HIREC. While our experiment showed that exposure to a constant long-term temperature increase can lead to long-term behavioural change, what remains unclear is how the timing, duration, and magnitude of warming and its discontents influence the onset and persistence of aggressive behaviour. Similarly, we cannot be certain as to how daily fluctuations in temperature, which are typical of all ecosystems, including tropical ones such as Lake Tanganyika^28,56^, would affect the persistence and intensity of aggression in ectotherms such as *J. ornatus*. We did, however, find that unintended short-term temperature fluctuations (increases) in our control treatment towards the end of the experiment (Supplemental Fig. S3) did not cause corresponding increases in aggressive behaviour in our study individuals. This finding supports our hypothesis that aggressive behaviour is driven more by long-term thermal fluctuations (months) than short-term ones (days to weeks). In our experiment, while the low temperature treatment group experienced an unintended temperature fluctuation briefly close to the end of the experiment, we did not observe a corresponding increase in aggressive behaviour in these fish. Clearly, answers to these questions will be necessary to better understand the response of wild populations of this and other cichlid species.

While our experiment explicitly tested individual fish behavioural responses to an artificial image of itself (similar size and reactions), which some have argued does not fully represent fish aggression perfectly^100–102^, aggressive intra-pair interactions do occur, with the larger individual typically being more dominant (Authors, personal observations). Because *Julidochromis ornatus* do not exhibit obvious sexual dimorphism (e.g., colour differences, mating tubercles), body size is an important determinant of parental behaviour. These observations correspond with natural observations of aggressive behaviours in *J. ornatus* where aggression is often targeted at conspecifics with the larger individuals generally being the aggressor^103^. When in breeding pairs, larger individuals tend to dominate and coerce their smaller partner into providing more parental care^60,80^. Hence, we would expect heightened aggression directed at a mate to lead to reduced fitness via mate mortality and reduced reproductive output. This notion raises the interesting question of whether the reduced reproductive output observed in a small subset of our study individuals exposed to a high temperature^27^ was a direct response of food limitation (i.e., a metabolic response), heightened aggression and competition for food (i.e., behavioural response), or both. Clearly, more work is needed to delineate the relative importance of these potential mechanisms.

In larger breeding groups with polygamy and cooperative breeding, the effect of temperature on behaviour would require further study, owing to the inherent complexities of sociality. For example, a similar study investigating temperature effects on fish behaviour, but in a social context, also observed a similar temperature-aggression relationship to our own^41^. Interestingly, the authors also found that subordinate individuals had lowered growth rates under higher temperature relative to the dominant individuals, which were unaffected^41^. Findings such as these highlight the need to explore how temperature-driven increases in aggression might affect social interactions, group structure, and reproductive output in *J. ornatus* and other cooperatively breeding species.

In conclusion, we cannot pretend that our simple, artificial experiment can allow us to predict with any certainty how continued warming and all of its discontents (e.g., reduced primary production)^28–30^ will drive behavioural change and fitness in tropical ectotherms such as *J. ornatus* in the wild. Even so, we do feel that our experiment offers important, novel insights that provide an excellent basis for future laboratory- and field-based research. Specifically, our findings indicate the potential for some tropical species to cope with warming and perhaps other forms of HIREC^28–30^ by adjusting their behaviour. To this end, we showed that individual aggressiveness in *J. ornatus*, a common cichlid endemic to Lake Tanganyika, increased when exposed to the warmer water temperature projected for Lake Tanganyika at the end of the 21^st^ century, with this behavioural adjustment being long-lasting (i.e., for the entirety of our 8-month experiment). Furthermore, our experiment offered a clue into the proximal mechanism underlying this behavioural shift as the most pronounced increases in aggression occurred in individuals in the poorest body condition (health). Knowing that long-lasting, climate-driven behavioural adjustments might be possible offers some optimism, given that tropical species are viewed as being especially vulnerable to climatic warming^4,9,20–22^. For example, the ability to become more aggressive could allow this species, as well as other endemic African cichlids with similar life-histories, to cope with the immediate effects of warming by improving access to critical resources such as breeding shelters and food^85–89^. Importantly, this temperature-induced increase in aggression may also come with costs, including increased predation risk or increased mate aggression that could potentially undermine reproductive success.

Given these tradeoffs, we are excited by the potential of future research endeavors aimed at determining whether heightened aggression serves as a net benefit or cost to individuals under increased thermal regimes. This question seems especially interesting in ecosystems such as Lake Tanganyika, which are also experiencing reduced energy production at the base of the food web due to climate warming^28–30^. Learning how water warming interacts with reduced food availability to drive individual behaviour, and in turn social and food web interactions, is an area in need of research, which could greatly benefit efforts to conserve the many unique species found in tropical ecosystems like the African Rift Lakes. Likewise, insight into whether environmentally-driven behavioural adjustments (e.g., increased aggression in response to warming) are truly adaptive and can be passed onto new generations through phenotypic selection and (or) temperature-induced epigenetic effects^18,19^, which has been implied in other non-behavioural studies^104,105^, seems especially important for understanding the ability of tropical organisms to cope with novel environmental change. Finally, owing to the many forms of HIREC that are simultaneously affecting the world’s aquatic and terrestrial ecosystems^1,3,7,106^, we encourage research that identifies linkages among environmental conditions, individual physiology, morphology, and behaviour^36^. Such knowledge could provide a much-needed platform to help us predict the response of broader population and social dynamics to independent and interacting forms of human-driven environmental change.

## Methods

### Study system and species

Lake Tanganyika is one of the oldest and largest freshwater lakes in the world, supporting approximately 10 million people from Burundi, the Democratic Republic of Congo (DRC), Tanzania, and Zambia^59^. During the past 1,500 years, Lake Tanganyika has had a stable climate with average temperatures remaining relatively constant until the early 1900s^56^. Since this time, temperature variation has increased (ranging 23.3 and 28.8 $^\circ$C)^61^, with the average temperature in both shallow and deeper regions of the lake also increasing^28,30,56^. While water temperatures in deep (> 100 m depth contour), relatively unproductive regions of the lake only increased by 0.2–0.3 $^\circ$C during the past century^28,30^, the rate of increase in shallower, historically productive waters has been about 1 $^\circ$C per 25 years since the mid-1960s, which approximates observed increases in air temperature^56^. Associated with these changes has been reduced primary production at the base of the food web, which is expected to limit food availability to secondary and tertiary consumers such as fish^28,29,58^.

Owing to its large size, great depth, old age, and historically stable tropical climate, Lake Tanganyika supports hundreds of endemic species, including fishes in the family Cichlidae^28,56,59,107,108^. Many species of these cichlids and the larger predator populations that they support are vital to the livelihoods of people in this region of the world as both a source of protein and a source of income^59,109,110^.

Our study species, *Julidochromis ornatus*, is a substrate-brooding cichlid endemic to Lake Tanganyika. *J. ornatus* individuals generally inhabit rocky shorelines in both the northern and southern sections of the lake^60,80^. *J. ornatus* exhibits substantial variation in its breeding (i.e., mating and social) system, which includes monogamy, polygamy, and cooperative breeding^60,80,111^. Even so, the formation of monogamous breeding pairs (i.e., pair-bonds) is the most common, occurring in the majority of shallow-water breeding groups and being twice as frequent as the next most observed breeding system^60^. Given that *J. ornatus* is commonly found in shallow areas of Lake Tanganyika, where water warming has been occurring rapidly since the mid-1960s^56^, and it is ecologically similar to many other small substrate-brooding cichlid species throughout the tropics^60^, we view it as a good indicator species for the many other cichlids inhabiting the lake.

### Experimental setup

The individuals used in our laboratory experiment were three generations removed from Lake Tanganyika. We received 200 small juveniles (about 3 months of age) from Reserve Stock Cichlids (East Chatham, NY, USA). During the setup phase of the experiment, the fish were housed at higher densities in 57-L glass aquaria, each containing multiple breeding shelters made of two 7 cm $\times$ 7 cm slate tiles. As part of a larger experiment investigating fish responses, such as metabolic rate, reproductive life-history to climate warming, we allowed these individuals to form pair-bonds ($n=72$ male-female pairs) on their own, which happened between 6–8 months of age. Once a pair-bond was formed, we randomly assigned that pair to a 57-L aquarium that had crushed coral substrate (CaribSea Florida Crushed Coral, Fort Pierce, FL, USA) and a single slate-tile breeding shelter. The aquaria were evenly and randomly distributed among four independent recirculating systems labelled A, B, C, and D ($n = 18$ pairs, one per aquarium, per recirculating system). Temperature was controlled using a single 500 W titanium aquarium heater (True Temp Titanium Heating System, JBJ, St. Charles, Missouri, USA) located in the sump reservoir of each recirculating system.

Following pair-bond formation, we continued to maintain all breeding pairs at 25.5 $^\circ$C, which is the mean annual water temperature of Lake Tanganyika during the 20^th^ century. We aimed to maintain all breeding pairs at 25–26 $^\circ$C until about 18 months of age (10–12 months of acclimation), at which time we began the experimental manipulation. This 25–26 $^\circ$C temperature range represents the mean annual water temperature of Lake Tanganyika during the 20^th^ century^56,58^. At 18-months of age, we began the experimental (temperature) manipulation, which lasted 8 months (until fish were 26 months of age). The use of a long (8-month) post-manipulation assessment period allowed us to overcome any potential short-term behavioural adjustments that might occur immediately after the manipulation.

Two sets of recirculating systems (A and C; low-temperature systems; n = 36 tanks) were used as controls with their mean temperature ($\pm 1$ standard deviation, SD) before and after manipulation being $25.1 \pm 0.5 \,^\circ$C and $25.1 \pm 0.5 \,^\circ$C, respectively (Supplementary Figs. S4, S5). By contrast, we raised the temperature in the other two recirculating systems (systems B and D; high-temperature systems; $n = 36$ tanks) over the course of two weeks in a linear manner (+ 2 $^\circ$C per week) until they reached ~ 29 $^\circ$C. The temperature was increased gradually over the course of each week in part due to the large volume of water in each recirculating system (approx. 1360 L), which ensured a slow rise in temperature. This temperature was determined by assuming that the 1 $^\circ$C increase in surface water temperature that occurred during the mid-1960s through 1990^56^ would continue through the remainder of the 21st century (until 2100). We feel justified in our use of this projected temperature, given that Lake Tanganyika surface water temperatures have already reached 28.8 $^\circ$C during summer months^56,61^ and the annual rate of air temperature increase in the region has only been accelerating through time^28,30,56^. The final mean ($\pm 1$ SD) temperatures observed our high-temperature systems (B and D) were $25.2 \pm 0.5 \,^\circ$C before temperature manipulation and $28.1 \pm 1.0 \,^\circ$C after manipulation through the conclusion of the experiment.

The temperature differences between the four recirculating systems through time (before and after temperature manipulation) were compared and tested using a 2-way ANOVA with system, time, and their interaction as predictive factors. This analysis confirmed that our experimental manipulation was successful. Specifically, it showed no significant differences in temperature among our systems before or after manipulation despite an unintended short-term temperature fluctuation in systems A and C near the end of the experiment (Supplementary Figs. S3 and S4). No significant differences existed between systems B and D (high temperature systems) before or after temperature manipulation either. However, they both differed from systems A and C (low temperature systems) after the temperature manipulation (Supplementary Fig. S4).

Physiochemical attributes were rigorously monitored throughout the experimental period. We measured temperature (nearest 0.01 $^\circ$C), dissolved oxygen concentration (nearest 0.01 mg/L), and dissolved oxygen saturation (nearest 0.1%) in each set of recirculating tanks continuously (every 30 min) with a YSI 5500D MultiDO Optical Monitoring and Control Instrument (Model 606927, Yellow Springs International, Ohio, USA), supplementing these observations with daily measurements from a ProPlus Multiparameter probe (Model 6050000, Yellow Springs International, Yellow Springs, Ohio, USA). We also measured pH, nitrate, nitrite, and ammonia weekly with API Freshwater Master Test Kits (State College, Pennsylvania, USA), conducting water changes as needed to keep conditions within acceptable levels (pH range = 7.2–8.5; nitrate < 10 ppm; nitrite < 0.1 ppm; ammonia < 0.1 ppm). We maintained high water-quality standards throughout the experimental period, with a temporary (< 2 months) reduction in ambient dissolved oxygen conditions in systems C (low-temperature system) and D (high-temperature system) to support another aspect of the project.

All rearing, euthanasia, and maintenance protocols described below followed approved Institutional Animal Care and Use Committee guidelines (IACUC protocol #2012A00000112, to SAL). All breeding pairs were reared with a 12:12 light:dark cycle, which closely approximate the light cycle near Lake Tanganyika, using high-intensity LED light strips located at the same distance above each tank. All individuals were fed cichlid pellets (Hikari Sinking Cichlid Gold Pellets: 45% crude protein, 5% crude fat, 2% crude fiber) daily at a standard ration of two pellets per fish each tank. Feeding occurred during morning hours, typically 0900–1200. While we could not ensure that the four pellets added to each tank daily were evenly shared between individuals within a tank, the fact that all individuals used in this study survived for at least 26 months suggests that partitioning of food occurred, with sufficient food existing to meet the routine metabolic needs of each individual.

### Behavioural assessment

We conducted behavioural trials when the individuals were about 9 months of age (1–3 months after pair-bond formation and ~9 months before the temperature manipulation in systems B and D), repeating the assessment ~ 26 months of age (~ 8 months after temperature was raised in systems B and D). The behavioural trials were conducted in the afternoon, after the daily feeding, in two 76-L glass aquaria (76.2 cm $\times$ 30.5 cm $\times$ 30.5 cm) using water from each individual’s originating recirculating system to minimize stress. We recorded temperature and dissolved oxygen saturation before and after each trial to ensure that they remained consistent during the trial (Z.X. Kua, unpublished data). Each behavioural test tank was divided into two sections that were separated by a removable barrier: 1/6 of the tank was designated as a holding area, with the other 5/6 of the tank being used as an exploratory arena used for additional behavioural trials (not reported herein). A high-definition video camera (Sony HDR-CX330) was mounted above each tank, as well as on a tripod beside each test tank, to record fish behaviour during the trials.

While mortality did occur in our experiment (Supplemental Table S1), we only included behavioural data from individuals with intact pair bonds. We tested both individuals from each breeding pair simultaneously (one individual per test aquarium), with breeding pairs from both treatments tested in a semi-randomized order. More specifically, we only tested breeding pairs from the same temperature treatment (same recirculation system) on any given day (maximum of 8 fish per day), with the breeding pairs chosen for testing being randomized within the recirculating systems for that temperature treatment. We randomized the order in which the two temperature treatments were selected for testing (on any given day). We conducted a 25% water change in each test aquarium after each trial, to reduce the influence of chemical cues (e.g., pheromones) on subsequently tested individuals.

Our behavioural assessment methods followed those of Schürch and Heg^50^, albeit slightly modified for our study species, in the form of a mirror to elicit aggressive response from the test subject. Specifically, each aggression trial was preceded by a 10-min acclimation period in the holding area, with the opaque (non-reflective) side of the mirrored partition facing the fish, before starting. The 10-min aggression trial began by flipping the partition for the test subject to face the mirror to elicit conspecific aggression. The observer either remaining distant and immobile during the experiment or left the room until the trial ended.

Because aggression can come in many forms^112^, we sought to record both overt (i.e., ramming, biting, tail-slap, and charging) and restrained (i.e., fin raising, lateral display, and head-down approach) aggressive displays (all trials were conducted or supervised by E.A.H., A.L.M., and Z.X.K.). We used JWatcher (version 1.0; www.jwatcher.ucla.edu) to tally the observed aggressive behaviours from the recorded videos, using well-described categories^112^. We calculated a total aggression score during each 10-min trial by summing the counts of both types of aggressive displays.

All videos were scored by a single person to eliminate inter-scorer bias. A subsequent blind rescoring of a subsample (n = 24; ~25%) of videos was conducted by Z.X.K. to verify accuracy, consistency, and avoid potential bias. Specifically, S.A.L. used a random number generator to select 24 trial videos for Z.X.K. to rescore while keeping the scorer completely blind to any information (e.g., identity, treatment, initial score) about each trial. Statistical comparisons confirmed that the video scoring was reliable and reproducible (Supplementary Table S2).

### Body size and condition

We measured the wet mass and total length of each individual prior to the first behavioural assessment (9 months of age) and at the conclusion of the experiment (26 months of age, at the time that we assessed aggression). These measurements were made because fish size and health have been shown to affect individual behaviour^36^. Thus, these measurements could potentially help us build better predictive models of aggression by considering them as possible covariates in our explanatory model of fish aggression.

Additionally, these measurements allowed us to calculate a measure of individual body condition, which can be viewed as a proxy for fish health^113^. To this end, we calculated a scaled-mass condition index (*cond*) following the method described by Peig and Green (2009)^113^. This method avoids potential biases associated with allometric growth by standardizing the condition of each individual by a fixed initial total length ($TL_0$; Eq. 1).

1 $$\begin{aligned} Condition = M_i[\frac{TL_0}{TL_i}]^{b_{SMA}} \end{aligned}$$

In this equation, the fixed length ($TL_0$) was determined by using the mean total length (*TL*; nearest 1mm) of our study population, with this mean generated from measurements taken at both 18 months and 26 months of age. $M_i$ and $TL_i$ are the wet body mass (nearest 0.1 g) and the *TL* of each individual (*i*), respectively. The scaling exponent was estimated by the standardized major axis (SMA) regression of *lnM* on *lnTL*, using all measurements at 18 and 26 months of age^113^.

### Statistical analyses

We used a mixed-modelling approach to assess differences in average *M*, *TL*, and *cond* at the start and end of the experiment. The fixed effects of temperature ($n=2$ levels, Low [25 $^\circ$C] vs. High [29 $^\circ$C]), time ($n=2$ levels, Pre [9 months] vs. Post [26 months]), system ($n=4$ levels, A, B, C, and D) and their interactions were assessed, with individual included as a random effect in the models. We used Tukey-adjusted least square means (LS means) to quantify differences for effect terms found significantly different. We used LS means instead of unadjusted means to account for uneven sample sizes between treatments^114^, which occurred due to mortalities during the course of the experiment. To avoid additional analytical complications with regard to sociality of our fish due to mortality, we only included fish which were housed in pairs in our statistical analyses.

We also sought to use a linear mixed-modelling approach to understand the factors driving variation in total aggression scores. Fixed effects assessed in the candidate set of predictive models included temperature treatment (Low vs. High), time (Pre vs. Post), and sex (Male vs. Female). All interactions among these fixed effects were also assessed.

Several covariates were also assessed for inclusion in our final predictive model. We included *M* (natural log-transformed) and *cond* as potential covariates. We also included a dummy variable behavioural test rank (order), as a potential covariate in our candidate models to account for potential effects of behavioural trial order on aggression. Behavioural test rank was the numeric order (1, 2, 3 or 4) of a behavioural trial relative to the 1, 2 or 3 other trials conducted in that tank during the same day. This assessment was necessary, given that only partial (25%) water changes occurred between trials, which could potentially have left behind pheromones that affected subsequent tests.

We assessed the need to include tank and individual as random effects in our final predictive model. Learning from likelihood ratio tests, however, that neither tank nor individual significantly improved the model fit when included (Supplementary Table S3), we did not include either in any of the candidate predictive models of total aggression. Thus, our final candidate predictive models only included fixed effects.

We used Akaike’s Information Criterion for small sample sizes (AICc) and AICc model weights to identify the most parsimonious predictive model of total aggression from our candidate model^115^. Models with $\Delta$AICc < 2 were considered equally plausible^115^. Following identification of the most parsimonious model, we used analysis of variance (ANOVA) to compare the effects of the included factors on total aggression. Those factors that were significant in our model were subjected to a Tukey-adjusted comparison of least squares means (LS means) to determine which treatments differed from one another. Similar to our tests of *M*, *TL*, and *cond*, we used LS means instead of unadjusted means to account for uneven sample sizes between treatments^114^.

All data used in our analyses were normal (Kolmogorov-Smirnov normality tests, all $p > {0.1}$), with an $\alpha$-value of 0.05 used to determine significance in all tests. We conducted all analyses using R 3.4.3^116^ with packages car^117^, lme4^118^, lsmeans^119^, emmeans^120^, MuMIn^121^, and nortest^122^.

## Supplementary information

Supplementary Information.

## Data Availability

The behavioural videos and data sets generated during and/or analyzed during the current study are available from the corresponding author on reasonable request.
