# Supplementary material for: Water warming increases aggression in a tropical fish
Source: Sci Rep. 2020 Nov 18;10:20107. doi: 10.1038/s41598-020-76780-1 (PMC7676273; doi:10.1038/s41598-020-76780-1)
Supplement: Supplementary file 1 — Supplementary Information. [file 41598_2020_76780_MOESM1_ESM.pdf]

Water warming increases aggression in a tropical fish

Zi Xun Kua[1,2], Ian M. Hamilton[3,4], Allison L. McLaughlin[1,5], Reed M. Brodnik[1,6], S. Conor Keitzer[1,7], Jake Gilliland[1,8], Elizabeth A. Hoskins[3], and Stuart A. Ludsin[1,3,*]

[1]Aquatic Ecology Laboratory, Department of Evolution, Ecology, and Organismal Biology, The Ohio State University, 1314 Kinnear Road, Columbus, OH 43212, USA
[2]Current: Department of Sustainable Resources Management, College of Environmental Science and Forestry, State University of New York, 1 Forestry Drive, Syracuse, NY 13210, USA
[3]Department of Evolution, Ecology, and Organismal Biology, The Ohio State University, 318 W 12th Avenue, Columbus, OH 43210, USA
[4]Department of Mathematics, The Ohio State University, 318 W 12th Avenue, Columbus, OH 43210, USA
[5]Current: Department of Biology, University of Kentucky, 101 T.H. Morgan Building, Lexington, KY 40506, USA
[6]Current: Chesapeake Biological Laboratory, University of Maryland Center for Environmental Science, 146 Williams St., Solomons, MD 20688, USA
[7]Current: Department of Natural Science, Tusculum University, Greenville, TN 37745, USA
[8]Current: Nationwide Children's Hospital, Columbus, OH 43205, USA
[*]email: ludsin.1@osu.edu; office: 614-292-1613; mobile: 614-795-7044; fax: 614-292-0181

ABSTRACT

Our understanding of how projected climatic warming will influence the world's biota remains largely speculative, owing to the many ways in which it can directly and indirectly affect individual phenotypes. Its impact is expected to be especially severe in the tropics, where organisms have evolved in more physically stable conditions relative to temperate ecosystems. Lake Tanganyika (eastern Africa) is one ecosystem experiencing rapid warming, yet our understanding of how its diverse assemblage of endemic species will respond is incomplete. Herein, we conducted a laboratory experiment to assess how anticipated future warming would affect the mirror-elicited aggressive behaviour of *Julidochromis ornatus*, a common endemic cichlid fish in Lake Tanganyika. Given linkages that have been established between temperature and individual behaviour in fish and other animals, we hypothesized that water warming would heighten average individual aggression. Our findings support this hypothesis, suggesting the potential for water warming to mediate behavioural phenotypic expression through negative effects associated with individual health (body condition). We ultimately discuss the implications of our findings for efforts aimed at understanding how continued climate warming will affect the ecology of Lake Tanganyika fishes and other tropical ectotherms.

Supplemental Materials

Table S1. Number of *Julidochromis ornatus* mortalities due to natural causes, including disease, injury, and jumping out of the tank. Live fish were tallied at the start of temperature manipulation (2014-09-01) and at the end of the experiment (2015-07-09). Time indicates the survey period. Temperature indicates temperature treatment: Low = 25.5°C; High = 29°C.

Time	Temperature	Live	Dead	Total
Pre	Low	53	19	72
	High	58	14	72
Post	Low	43	29	72
	High	36	36	72

Table S2. Linear regression test statistics comparing original score counts and blind rescored counts of aggression trial videos with *Julidochromis ornatus*. Blind recounts of a subsample (n = 24; 25%) of videos were conducted to test for consistency and bias of the original scorer (Z.X.K.). Videos for rescoring were selected by S.A.L. using a random number generator. These videos consisted of 12 trials from the low temperature treatment (system A = 8; system C = 4) and 12 trials from the high temperature treatment (system B = 5; system D = 7). All videos were re-labeled randomly to ensure that Z.X.K. was blind to which treatments the videos originated. The slope and intercept of the least-squared regression lines describing the relationship between our original counts and recounts of aggression scores did not differ from a 1:1 line with an intercept of 0 (95% confidence interval of each slope and each intercept encompassed the value of 1 and 0, respectively).

Response variable	r	Model attribute	Parameter estimate	95% Cl of estimate
Total Aggression	0.92	Slope	1.04	[0.84, 1.24]
		Intercept	-3.21	[-43.98, 37.56]
Overt Aggression	0.84	Slope	1.08	[0.75, 1.42]
		Intercept	8.84	[-13.95, 31.63]
Restrained Aggression	0.82	Slope	0.78	[0.56, 1.01]
		Intercept	23.18	[-8.53, 54.88]

Table S3. Likelihood-ratio test (LRT) results used to compare the linear models for individual *Julidochromis ornatus* aggression, to determine if "Tank" and (or) individual (ID) should be included as random effects. Each model was compared with the model above in the table. "Aggression" indicates aggression scores calculated from behavioural assessment. "Tank" indicates aquaria housing each fish pair. "Variables" indicates the main factors used to predict fish aggression scores (i.e., temperature, time, and condition). Pr(>Chisq) values greater than 0.05 indicate no significant difference. Akaike Information Criterion (AIC), Bayesian Information Criterion (BIC), and log likelihood (logLik) were used to estimate model fit. Degrees of freedom (Df) indicate model complexity.

<table>
<tr><th></th><th>npar</th><th>AIC</th><th>BIC</th><th>logLik</th><th>deviance</th><th>Chisq</th><th>Df</th><th>Pr(>Chisq)</th></tr>
<tr><td>Null: Aggression = Variables</td><td>12</td><td>1132.19</td><td>1163.69</td><td>-554.09</td><td>1108.19</td><td>NA</td><td>NA</td><td>NA</td></tr>
<tr><td>Tank: Aggression = Variables + (1|Tank)</td><td>13</td><td>1133.84</td><td>1167.96</td><td>-553.92</td><td>1107.84</td><td>0.35</td><td>1</td><td>0.55</td></tr>
<tr><td>ID: Aggression = Variables + (1|ID)</td><td>13</td><td>1134.19</td><td>1168.31</td><td>-554.09</td><td>1108.19</td><td>0.00</td><td>0</td><td>1.00</td></tr>
<tr><td>Global: Aggression = Variables + (1|Tank/ID)</td><td>14</td><td>1135.84</td><td>1172.59</td><td>-553.92</td><td>1107.84</td><td>0.35</td><td>1</td><td>0.55</td></tr>
</table>

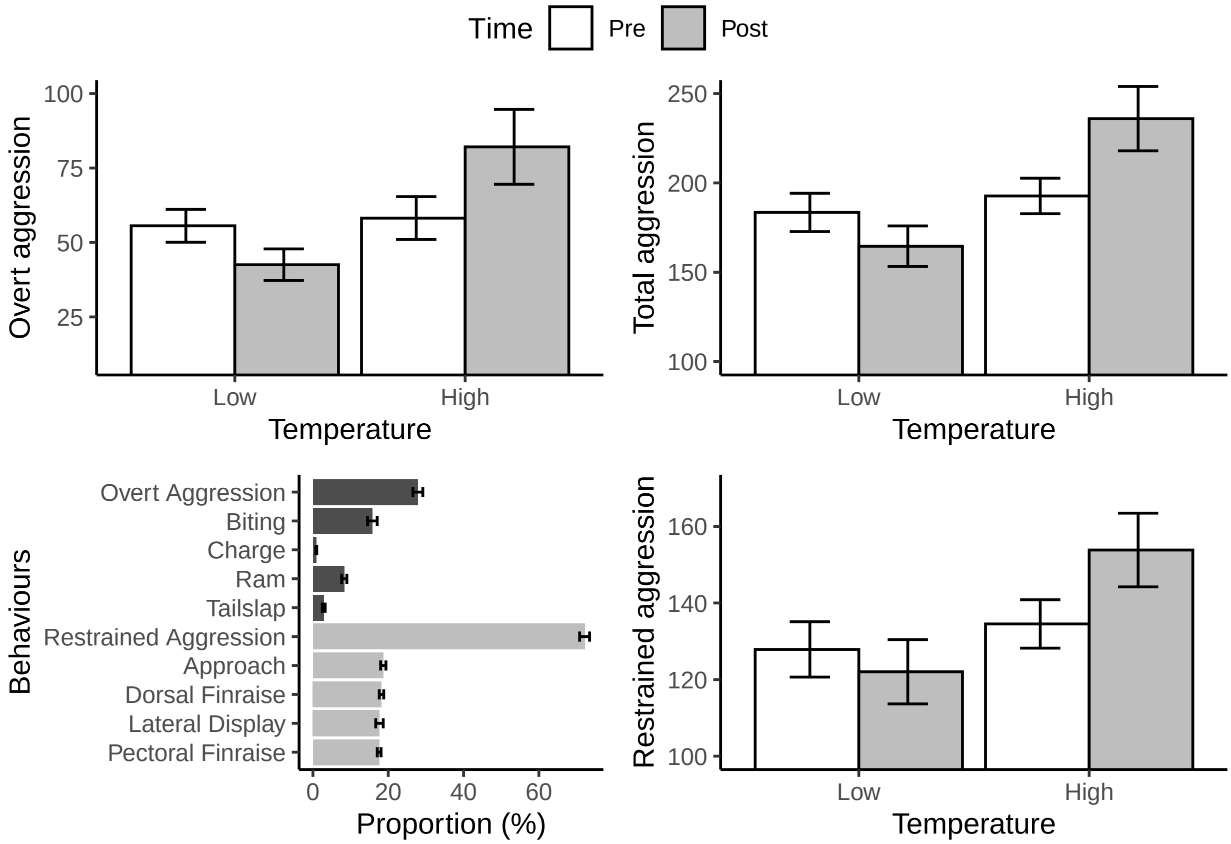

Figure S1. Breakdown of aggression scores observed from the aggression trials conducted with *Julidochromis ornatus*. Top left: overt aggression scores for each time period by temperature treatment. Top right: total aggression scores for each time period by temperature treatment. Bottom left: mean proportion of each recorded aggressive behaviour in relation to total aggression. Bottom right: restrained aggression scores for each time period by temperature treatment. While all individuals were subjected to 25°C (Temperature = Low) prior to the start of the experiment (Time = Pre; 9 to 18 months), temperature was raised to 29°C during 18-26 months of the experiment (Time = Post) in half of the tanks (Temperature = High) whereas it remained unchanged in the other half of the tanks (i.e., Temperature = Low). The mean (bars) and errors (1 standard error) for each figure are presented.

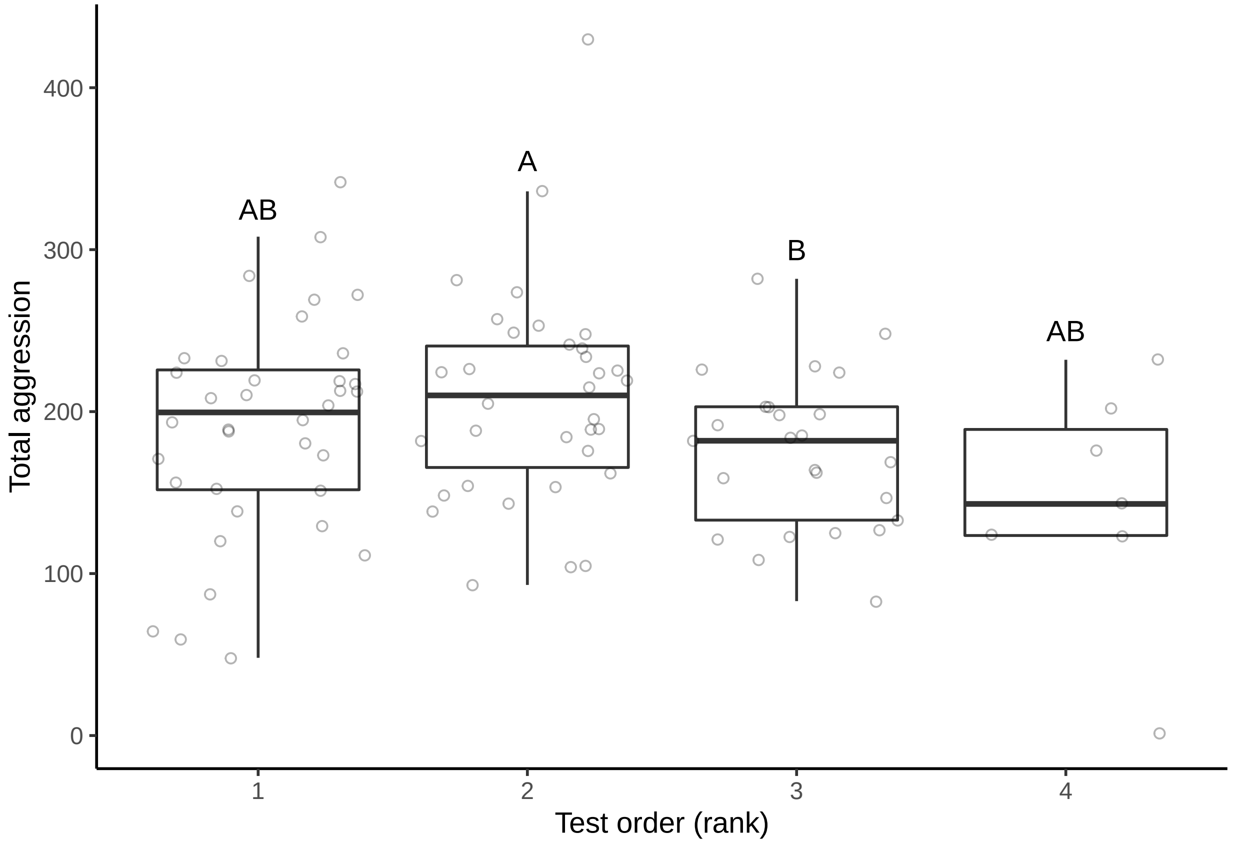

Figure S2. Boxplot of individual cichlid total aggression score versus test rank. Behavioural test rank was the numeric order (1, 2, 3 or 4) of a behavioural trial relative to the 1, 2 or 3 other trials conducted in that tank during that same day. This assessment was necessary, given that only partial (25%) water changes occurred between trials, which could potentially have left behind pheromones that affected subsequent tests. Boxes indicate interquartile ranges with the center line indicating median. Whiskers indicate the minimum and maximum while points indicate outliers (lesser or greater than Q1 or Q3 + 1.5 IQR). Boxes sharing a letter do not significantly differ (Tukey-adjusted *LSMeans* comparisons; $p < 0.05$). Letters were obtained from testing residuals of the most parsimonious model which accounts for the confounding effects of other variables i.e., temperature. time period, size, and condition. Note, the low sample size in test rank = 4 indicates four trials were rarely conducted in the same day.

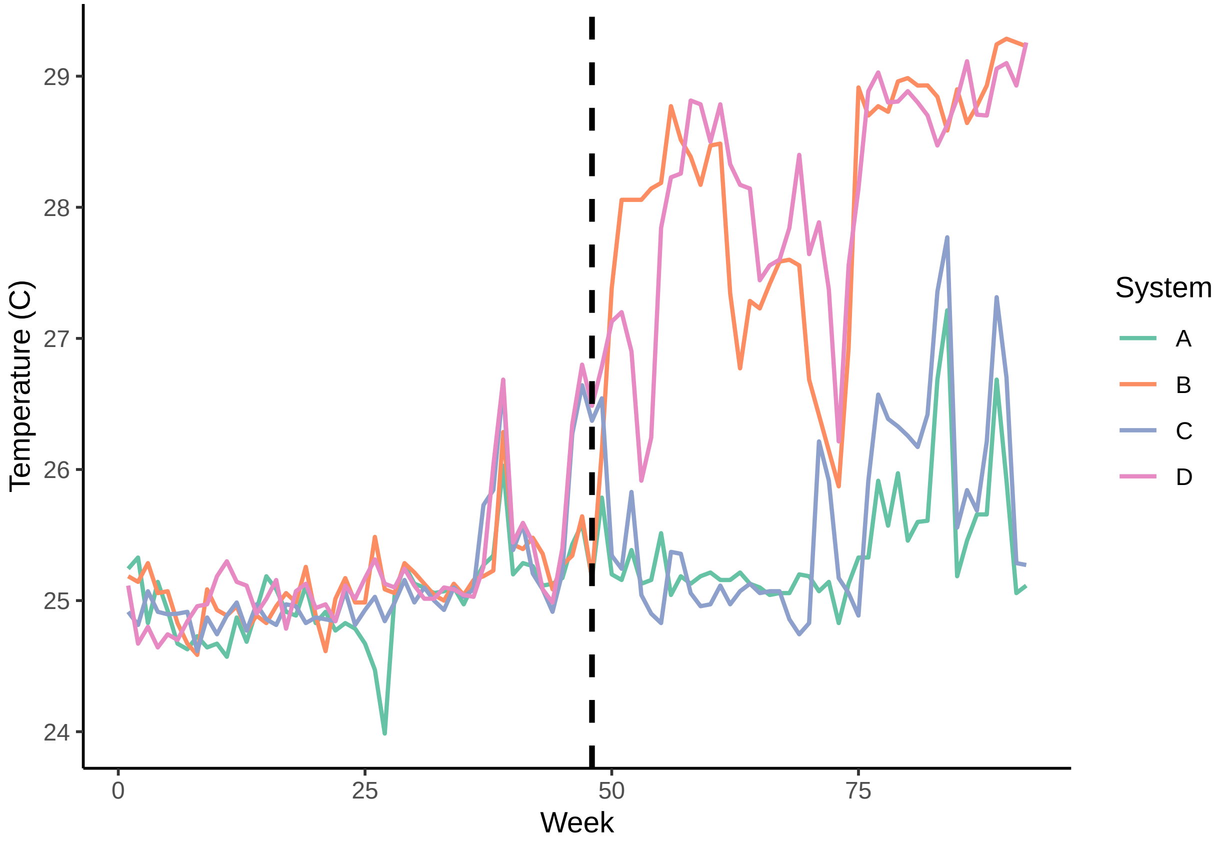

Figure S3. Time series of weekly average temperature for each recirculating system throughout the experiment. The vertical dashed line indicates start of the temperature manipulation in systems B and D. The "Pre" experimental period occurs left of the dashed line, with the "Post" experimental period occurring after.

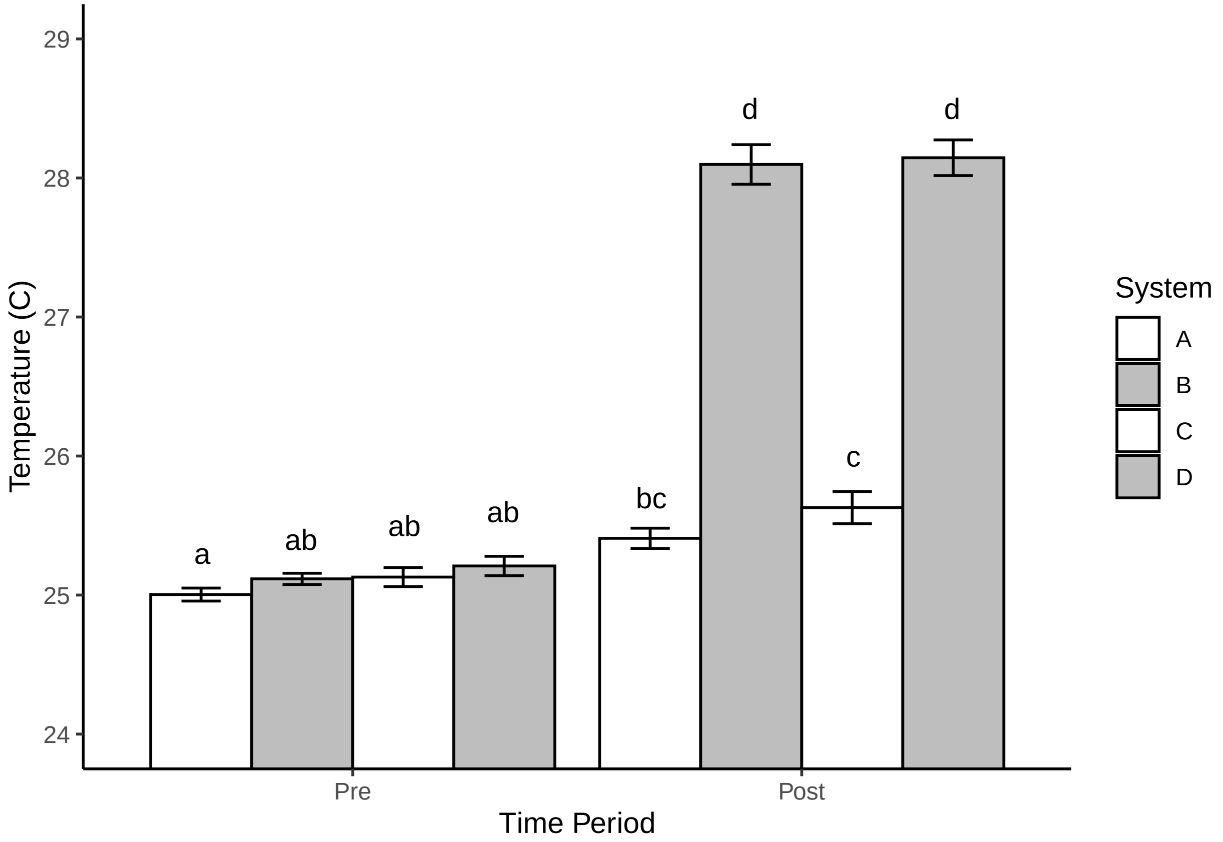

Figure S4. Average temperature of all four recirculating systems by time period, i.e., before ("Pre") and after ("Post") temperature manipulation. Temperatures in systems A and C were kept around 25-26°C throughout the whole experiment, whereas temperatures in systems B and D were raised from around 25°C after 6 months of acclimation to approximately 29°C gradually over a two-week period. The mean (bars) and associated error (±1 SE) of weekly temperature in each system are presented. Systems sharing a letter do not significantly differ (Tukey HSD test; $p < 0.05$).

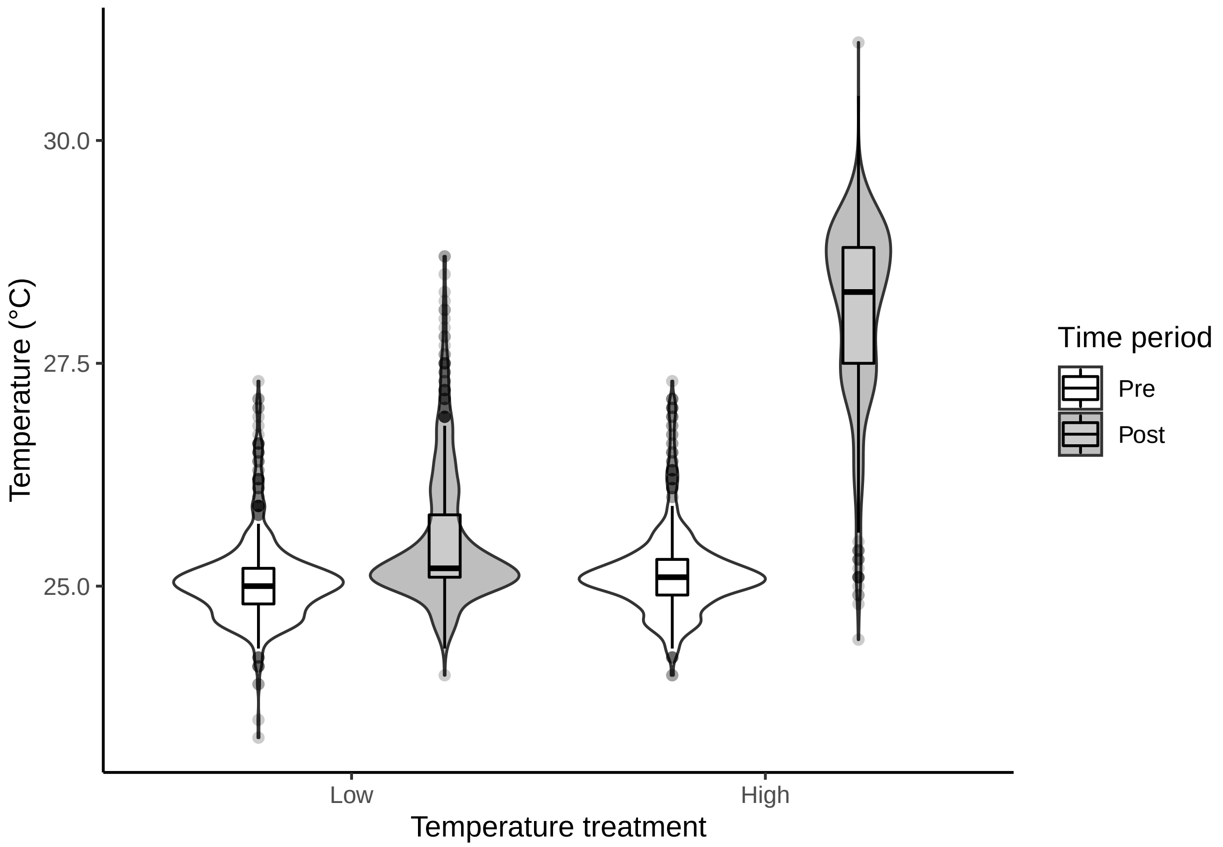

Figure S5. Combination of violin plots and boxplots of daily average of water temperature measurements in each treatment. All tanks were held to ~ 25-26°C (Temperature = Low) prior to the start of the experiment (Time = Pre; 9 to 18 months). Temperature was raised to 29°C during 18-26 months of the experiment (Time = Post) in half of the tanks (Temperature = High), whereas it remained unchanged in the other half of the tanks (i.e., Temperature = Low). Horizontal lines on the boxplots represent the median; whiskers represent the "minimum" (Q1 - 1.5 IQR) and "maximum" (Q3 + 1.5 IQR) value; points represent statistical outliers.
